# Rhythmic performance in hypokinetic dysarthria: Relationship between reading, spontaneous speech and diadochokinetic tasks

**DOI:** 10.1016/j.jcomdis.2018.02.005

**Published:** 2018

**Authors:** Anja Lowit, Agata Marchetti, Stephen Corson, Anja Kuschmann

**Affiliations:** a School of Psychological Sciences and Health, Graham Hills Building, Strathclyde University, 40 George Street, Glasgow G1 1QE Scotland, United Kingdom; b School of Psychological Sciences and Health, Strathclyde University, Psychological Sciences and Health, United Kingdom; c Dept. of Mathematics and Statistics, Strathclyde University, United Kingdom

**Keywords:** Rhythm, Dysarthria, Parkinson's disease, Spontaneous speech, DDK

## Abstract

• We investigated speech rhythm in people with Parkinson’s Disease (PwPD) and controls.
• Even mildly affected PwPD differed from controls in their rhythmic performance.
• PwPD showed less difference between reading and spontaneous speech.
• Spontaneous speech highlighted more differences between speakers than reading.
• DDK performance did not relate to rhythmic behaviour in connected speech.

## 1 Introduction

Hypokinetic dysarthria is a motor speech disorder associated with Parkinson’s Disease (PD), which accounts for around 8% of all dysarthrias (Duffy, 2013). It arises from a reduction in the mobility of movements and can manifest across all levels of speech production, including the respiratory, phonatory, resonatory, articulatory and prosodic system. People with PD (PwPD) often suffer from a lack of respiratory support (Mehanna & Jankovic, 2010; Moustapha et al., 2013), as well as phonatory problems which are most commonly characterised by a drop in volume (Ramig, Sapir, Fox, & Countryman, 2001; Tanaka, Nishio, & Niimi, 2011). Speech intelligibility is further impacted by changes in the precision of articulatory movements for vowels and consonants alike (e.g. Bunton & Weismer, 2001;McAuliffe, Ward, & Murdoch, 2006; Sapir, Spielman, Ramig, Story, & Fox, 2007; Skodda, Visser, & Schlegel, 2011; Tykalova, Rusz, Klempir, Cmejla, & Ruzicka, 2017).

Prosodic impairment is also a frequent symptom of PD. Prosody forms part of the suprasegmental features of speech and has traditionally been divided into phonetic and linguistic attributes. The phonetic attributes are defined as the parameters of F0, intensity, quantity/duration and silence when measured acoustically, or pitch, loudness, length, and pause in their perceptual forms (Bolinger, 1989). Modulation of these attributes leads to the linguistic components of prosody − intonation, stress, tempo, rhythm, and pause (Couper-Kuhlen, 1986), which are employed by speakers to generate or disambiguate meaning, draw listeners’ attention to important parts of the message and structure their discourse (Couper-Kuhlen, 1986; Cutler, Dahan, & van Donselaar, 1997; Laver, 1994). In addition to the reductions in volume referred to above, the PD literature reports deficits in pitch and intonation production (Ma, Whitehill, & So, 2010; Lowit & Kuschmann, 2012; Skodda, Visser, & Schlegel, 2008; Tykalova, Rusz, Cmejla, Ruzickova, & Ruzicka, 2014). Changes in movement rate have also been reported, both in speech and non-speech tasks. With regard to the latter, differences in diadochokinetic (DDK) task performance are frequently noted, although performance appears to be rather variable across different speakers. The literature thus describes unimpaired, reduced as well as faster repetition rates, in addition to an increase in the variability of syllable duration (Ackermann, Konczak, & Hertrich, 1997; Rusz, Hlavnicka, Cmejla, & Ruzicka, 2015; Skodda, Flasskamp, & Schlegel, 2010; Tjaden & Watling, 2003). These findings are mirrored in investigations of rate during speech tasks, i.e. investigations of speech and articulation rate have resulted in findings of similar, slower or faster rates in PwPD compared to their healthy controls (Hlavnička et al., 2017; Kim, Kent, & Weismer, 2011; Lowit, Dobinson, Timmins, Howell, & Kröger, 2010; Skodda & Schlegel, 2008). Number and duration of pauses are also reported to be higher than is observed in unimpaired speakers (Harel, Cannizzaro, Cohen, Reilly, & Snyder, 2004; Lowit, Brendel, Dobinson, & Howell, 2006; Rosen, Kent, Delaney, & Duffy, 2006; Skodda & Schlegel, 2008). More recently, rhythm disturbances have been added to the list of potential prosodic changes experienced by PwPD. These disturbances have been described for syllable repetitions (Skodda, Flasskamp et al., 2010; Skodda, Grönheit, & Schlegel, 2010), and connected speech (Liss et al., 2009). In addition, Späth et al. (2016) report that PwPD were less able to copy a rhythmical model during an entrainment task than healthy controls, and Grahn and Brett (2009) found that this speaker group was less able to process rhythmic tone sequences.

There has been a growing recognition of the central role that prosody plays for intelligibility and naturalness in speakers with PD as well as other types of dysarthria, which has made this area a growing focus for clinical research. Researchers have tried to identify how prosody might be affected across different types of dysarthria, what the effect of such impairment might be on the listener, and consequently how communication efficiency might be improved with the help of prosody. Rate and pause characteristics have already been referred to above. Stress has also been researched frequently (e.g. Lowit, Kuschmann, MacLeod, Schaeffler, & Mennen, 2010; Schalling, Hammarberg, & Hartelius, 2007; Schalling & Hartelius, 2004). On the other hand, aspects such as intonation and rhythm have not been investigated to the same extent, largely due to a lack of reliable methodologies to study them. For example, authors have been highlighting difficulties in dysarthria with the production of intonation for some time (Leuschel & Docherty, 1996; Ma et al., 2010; Robin, Klouda, & Hug, 1991; Schalling et al., 2007; Schalling & Hartelius, 2004; Skodda, Rinsche, & Schlegel, 2009). Yet this area has not been researched in any structured way until more recently when the autosegmental-metrical (AM) framework (Pierrehumbert, 1980) was adopted into clinical research (Kent & Kim, 2003; Kuschmann & Lowit, 2012; Kuschmann, Lowit, Miller, & Mennen, 2012; Kuschmann, Miller, Lowit, & Mennen, 2011; Lowit & Kuschmann, 2012; Lowit, Kuschmann, & Kavanagh, 2014; Mennen, Schaeffler, Watt, & Miller, 2008).

Research on rhythm follows a comparable pattern, i.e. although the presence of rhythmic changes or disturbed rhythm have been reported since the early characterization of dysarthria (cf. Darley et al., 1969a, Darley et al., 1969b), publications on this feature have noticeably increased in volume since the development of quantifiable measures in the form of acoustic rhythm metrics. These metrics were originally devised for cross-linguistic phonetic purposes, i.e. to differentiate languages with different rhythmic patterns from each other in a reliable way (see e.g. Ramus, Nespor, & Mehler, 1999; White & Mattys, 2007). However, they have also been applied successfully in the characterisation of impaired performance patterns across a number of dysarthria types.

All rhythm metrics are based on inter-relationships of either vowel, consonant or syllable durations, i.e. they might report on the proportion of vowels in the sample (%V, Ramus et al., 1999), simple measures of durational variability such as the standard deviation of vowels (ΔV) or consonants (ΔC, Ramus et al., 1999) or the coefficient of variation of either of these segments (VarcoV & VarcoC, White & Mattys, 2007). Others capture how durations differ from one vowel or consonant to the next (pairwise variability index (PVI), Low, Grabe, & Nolan, 2000). In addition, some measures focus on the durational variability of syllables (VarcoVC, nPVI_VC, rPVI_VC, Liss et al., 2009), or inter-stress intervals (ISI, Hartelius, Runmarker, Andersen, & Nord, 2000). They work on the premise that variability of any of these measurement units will be different between stress-timed and syllable timed languages. These assumptions are largely based on the phonetic and phonological properties of these languages. Two main principles were proposed by Dauer (1983) in this respect: (1) stress-timed languages have a greater variety of syllable types, in particular, they contain more consonant clusters than syllable-timed languages; and (2) stress-timed languages include a reduced quality of unstressed vowels (often limited to schwa), which are shortened significantly (cf. the first syllable in “potato” or the second and third in “elephant”) or omitted altogether (cf. Peter is coming − Peter’s coming). This means that the proportion of vowels (%V) in a syllable time language is often higher than in a stress-timed language, as less reduction takes place. On the other hand, the presence of consonant clusters as well as singletons, and the alternation between full and unstressed vowels typical for stress-timed languages results in a higher variability of duration of these segments, thus higher ΔV and ΔC values. The Varco measures are based on the same principle but also control for speech rate which was shown to impact on the ΔV and ΔC results (White & Mattys, 2007). The PVI measures take a slightly different approach and investigate the alternation in segment length in a pairwise fashion rather than as a global measure of variability across the whole sample. The sequence “**Pe**ter **runs** to **Zan**zi**bar**” which includes a periodic alternation of long and short syllables thus attracts higher PVI values for vowel duration than “The **e**lephant is a **large mammal**”, which consists of multiple sequences of short (“(e)lephant is a”) and long (“large mammal”) vowels. The same principal applies to the syllabic PVI measures, i.e. stress-timed languages that include consonant clusters and long versus single consonants and reduced vowel syllables will have a higher variability in syllable duration than syllable timed languages that consist of mainly single consonants and relatively equal length vowels.

The differences between stress- and syllable timed languages described above are similar to the phonetic disturbances that have been reported for dysarthric speech. For example, changes to singleton consonants or reductions of consonant clusters could have an impact on the variability of consonant durations. Similarly, problems with vowel production, such as reducing full vowels to more centralized, shorter vowels, or lengthening normally reduced vowels, would impact on vowel as well as syllable duration patterns. On this basis, the rhythm metrics described above should also be sensitive to changes in timing resulting from a neurogenic speech disturbance such as dysarthria. This was confirmed by a number of studies focusing on ataxic dysarthria, which has clear associations with rhythmic problems due to the underlying cerebellar impairment leading to poor coordination of speech movements (Kent et al., 2000; Schalling & Hartelius, 2004; Sidtis, Ahn, Gomez, & Sidtis, 2011). These studies found that a number of the above rhythm metrics were able to highlight differences in rhythmic performance between speakers with ataxia and healthy controls (Hartelius et al., 2000; Henrich, Lowit, Schalling, & Mennen, 2006). The studies also showed that the impaired speaker group tended to have a more syllable timed rhythm, which fitted well with the perceptual concepts of equalized stress and “scanning” speech associated with ataxic dysarthria.

Following on from this research, Liss et al. (2009) were the first to also apply the metrics to a wider range of dysarthria types, namely hypokinetic, hyperkinetic, flaccid-spastic, and ataxic dysarthria. They investigated a large number of different rhythm metrics and found that all types of dysarthria could be distinguished from healthy adults and to some degree from each other on the basis of a combination of different rhythm metrics focusing on vowel, consonant and/or syllable duration. Similar results were reported by Lowit (2014) in a study on individuals with ataxic and hypokinetic dysarthria. Studies to date thus indicate that rhythm metrics are promising tools to detect presence and possibly types of dysarthria, and could thus potentially function as diagnostic as well as outcome measures.

One yet unresolved issue in the cross-linguistic as well as clinical literature is the question to what degree these rhythm metrics actually reflect rhythm as opposed to simply speech timing, given their exclusive focus on segment duration. There have therefore been calls to also consider other speech parameters in the characterization of rhythm (Arvaniti, 2009; Nolan & Jeon, 2014). For example, Arvaniti (2009) calls for a return to the original concept of linking rhythm to stress, which is a function of not only duration but also pitch and intensity. Tilsen and Arvaniti (2013) subsequently demonstrated that a number of rhythmically distinct languages could be differentiated on the basis of their amplitude envelopes. Lowit (2014) furthermore suggested that intonation, in particular phrasing, can have an impact on the rhythmic performance of speakers with dysarthria. Whilst there is thus evidence that the currently available metrics cannot fully capture speech rhythm, no viable integrated, multimodal model of rhythm has yet been developed that considers the range of parameters that contribute to the perception of rhythm, and that could be reliably applied to clinical data at this moment. On the other hand, from a clinical perspective, there remain a number of questions to be explored with the current timing based models. In particular, given the small number of studies performed to date, there is value in conducting further research into whether one can confidently accept these methods into the battery of tools currently used in disordered speech research. This paper focuses on three issues that require further investigation to answer this question.

First of all, it is currently unknown to what degree the rhythm metrics^1^ are able to identify performance changes in speaker groups with a milder form of dysarthria. Both Liss et al.’s (2009) and Lowit’s (2014) studies mostly incorporated speakers of moderate to severe dysarthria. Henrich et al.’s (2006) study did include speakers with mild dysarthria and found that some of them performed within the control range. However, because they used a smaller number of rhythm metrics compared to Liss et al. (2009) and Lowit (2014) no conclusions can be drawn whether this finding was due to their particular set of measures not being sensitive to the speakers’ performance change which others might have detected, or whether these speakers simply did not show any impairment in this domain yet. Further research is thus necessary to establish the sensitivity of acoustic rhythm metrics to speakers with milder degrees of dysarthria.

Secondly, most rhythm research so far has used controlled read speech as the basis for analysis. This is due to the fact that the rhythmic structure of an utterance is highly dependent on its phonetic make-up. For example, Low et al. (2000) demonstrated that the results of their rhythm metrics depended on the number of full versus unstressed vowels contained in a given utterance (e.g. “**Bethatelunch**” vs “She had **af**ter**noontea**”). Such variables can obviously not be controlled for in spontaneous speech. Whilst Tilsen and Arvaniti (2013) were able to demonstrate differences between languages based on read as well as spontaneous speech, they caution that findings from the analysis of read speech cannot be generalized to conversational speech. In clinical research, it is well known that there are differences between speech performances in structured and more naturalistic, spontaneous tasks (Brown & Docherty, 1995; Kent, Kent, Rosenbek, Vorperian, & Weismer, 1997; Kuschmann & Lowit, 2015; Leuschel & Docherty, 1996, van Lancker Sidtis, Cameron, & Sidtis, 2012), and it is therefore advisable to capture patients’ naturalistic speech performance as part of a holistic assessment. In view of Tilsen and Arvaniti’s (2013) findings, it is therefore important to investigate whether uncontrolled, spontaneous speech samples can be used as the basis for rhythm analysis in clinical populations, and whether results differ in any way to those previously reported for these speaker groups.

Finally, the increasing evidence that a wide range of speakers with dysarthria are affected in their rhythm production suggests that this issue might have to be considered in the diagnosis of these speakers as well as in the planning of effective management plans. This necessitates the availability of easily applicable measurement tools. Unfortunately, the complexities associated with analyzing speech samples to calculate currently available rhythm metrics means that these approaches are not suitable for use in clinical practice at the moment. A potential alternative to measuring speech rhythm comes in the form of diadochokinetic (DDK) tasks. Whilst these maximum performance tests are generally applied to gather information on the speed of articulatory movement, they are also frequently evaluated in relation to the regularity of this movement, and thus their rhythm (Kent, 2015; Lowit, 2014; Skodda, 2011; van Brenk & Lowit, 2012). However, there is an ongoing debate about the validity of DDK tasks in reflecting speech behaviour (see Maas (2017) and Kent (2015) for a review), and Ziegler and colleagues have been arguing for some time that non-speech and speech tasks use different underlying control mechanisms and impose different task demands (Staiger, Schölderle, Brendel, Bötzel, & Ziegler, 2017; Staiger, Schölderle, Brendel, & Ziegler, 2017; Ziegler, 2002). Their most recent research was based on the analysis of 130 speakers with motor speech disorders and 130 healthy controls, performing speech, speech-like and nonspeech tasks. Kent (2015) adds to this argument with an extensive review of the disordered speech literature, and concludes that the value of DDK tasks in the assessment of dysarthria remains controversial. On the other hand, Skodda, Flasskamp et al. (2010), Skodda, Grönheit et al. (2010) claim similarities between disturbances in the rate of syllable repetition and the rhythm and rate acceleration observed in connected speech in their PD speakers and hypothesized that the performance across the two tasks reflected a similar pathophysiology. If their results were replicated and it could be demonstrated that DDK results reflect a similar form or degree of timing difficulties as those identified in a detailed speech rhythm analysis, then they could indeed represent a viable alternative for clinical practice. This area therefore also warrants further investigation.

This study aimed to address the above mentioned knowledge gaps by comparing the rhythmic performance of speakers with mild dysarthria and healthy controls in reading aloud and spontaneous speech, and to relate these results to their DDK performance. Given the paucity of investigations into disordered speech rhythm in general, and the absence of any information on rhythm in spontaneous speech in this speaker group our study looked at as wide a range of rhythm metrics as possible within the limitations of the statistical analysis in order to capture potential differences. The investigation specifically addressed the following questions:

1. Are acoustic rhythm metrics sensitive to articulatory change in speakers with mild dysarthria?
2. Is it possible to detect such changes in reading aloud as well as spontaneous speech, and are similar metrics implicated in both tasks?
3. What is the relationship between rhythmic performance in reading aloud, spontaneous speech and DDK tasks in speakers with dysarthria and healthy controls?

Based on reports of segmental and prosodic changes in speech production even in PwPD with mild severity of impairment, and the sensitivity of the rhythm metrics to small differences in speech production as observed across different languages, we hypothesized that our analysis should be able to highlighted differences between our PwPD and healthy control speaker group. In relation to the second research question, Tilsen and Arvaniti (2013) were able to replicate their findings for reading aloud with a conversational speech sample, and we thus anticipated that the same should be possible in our disordered speaker group. Finally, we predicted that there would be little direct relationship between DDK and connected speech performance in relation to rhythm based on most previous research. However, as explained above, the potential clinical benefits of finding a link between the two areas meant that this question needed further investigation.

## 2 Methodology

### 2.1 Participants

In order to find a homogeneous sample of speakers with mild dysarthria, this study used data that had previously been collected for other purposes from 42 people with Parkinson’s Disease (PwPD) and 20 healthy control participants (Brendel, Lowit, & Howell, 2004; Lowit et al., 2006; Lowit, Dobinson et al., 2010). This study had received ethical approval from all participating Health Board Research Ethics Committees. Inclusion criteria for the original study included a diagnosis of idiopathic PD as well as hypokinetic dysarthria as confirmed by the referring speech and language pathologist (SLP), no history of other medical problems or speech and language difficulties, and adequate cognitive, visual and hearing abilities to complete the task. In addition, all participants were native British English speakers and were older than 50 years.

A variety of speech tasks had been collected for the initial study, including maximum performance tasks, a phonetically balanced reading passage (Lowit et al., 2006), and a conversational sample about a holiday. Speaker selection for the current study was based on the intelligibility scores collected for the reading passage. Intelligibility scores had been derived through Direct Magnitude Estimation (Weismer & Laures, 2002; Whitehill, Lee, & Chun, 2002). This method requires listeners to judge a sample against a “standard”, which is given a score of 100. In our case the standard represented a PwPD with moderate dysarthria (as agreed by 5 trained listeners). Listeners then assign scores to the samples to be rated in multiples of how much more or less intelligible than the standard an individual was perceived to be. Scores below 100 thus indicated a moderate to severe dysarthria, whereas those of 100 and above reflected an increasing level of intelligibility up to normal speech. For example, 50 would be half as intelligible as the standard and 200 twice as intelligible. Each speaker’s score as provided here represents an aggregate of individual results from ten listeners (final year SLP students), who were familiar with dysarthria and had been trained in the DME procedure on data from PwPD that were not part of the study at the time. For the DME procedure, the speech samples had been randomised across all speakers and presented in groups of five (standard plus four samples for rating), i.e. listeners heard the standard, rated four samples, then heard the standard again and rated another four samples, etc. Each sample was only listened to once. Based on these intelligibility scores, the ten most mildly impaired PwPD were selected from the database, and matched with ten healthy control speakers for age and gender.

Table 1 provides information on the selected participants. All PwPD happened to be male, and their mean age was 68.2 years (SD = 5.75 years). The matched control speakers were also all male, with a mean age of 68.1 years and an SD of 5.63 years. The control group mean for intelligibility was 703, with an SD of 100 and a range of 538–850. As a reflection of the mild level of hypokinetic dysarthria, the PD group had a mean intelligibility score of 589, with an SD of 111 and a range of 438–748. The majority of the PwPD intelligibility scores were thus within the range for the controls, with only three speakers falling within one standard deviation of the minimum control value. The PwPD’s speech problems ranged from changes in loudness level or voice quality only to mild articulation impairment.

**Table 1:** Participant Information.

Participant	Age	Gender	Intelligibility	Time since diagnosis (years)	Medication
**PD1**	66	M	668	4	Sinemet, Sinemet CR, Entacapone
**PD2**	71	M	748	3	Madopar
**PD3**	75	M	486	6	Madopar
**PD4**	62	M	675	6	Bezhexol, Co-Codamol, Amlodipine, Bendropfluazide, Sinemet Plus, Ropinirole
**PD5**	67	M	533	11	Sinemet, Selegiline, Pergolide, Entacapone
**PD6**	62	M	438	10	Amantadine, Sinemet, Ropinirole
**PD7**	71	M	464	10	Sinemet, Domperidone
**PD8**	71	M	581	11	Selegiline, Sinemet CR, Madopar, Entacapone
**PD9**	60	M	725	5	Pramipexole, Finasteride, Co-Codamol, Quinine bisulphate
**PD10**	77	M	568	6	Madopar
					
**CON1**	64	M	701		
**CON2**	70	M	850		
**CON3**	74	M	592		
**CON4**	62	M	538		
**CON5**	66	M	766		
**CON6**	77	M	777		
**CON7**	69	M	583		
**CON8**	59	M	752		
**CON9**	67	M	719		
**CON10**	73	M	753		

PD = speaker with Parkinson’s Disease; CON = Control speaker. Intelligibility values reflect the Direct Magnitude Estimation (DME) score, where 100 represents a PD speaker with moderate dysarthria. Increasing scores correspond to higher levels of intelligibility.

### 2.2 Speech materials and data collection methods

Data were collected in a quiet location at the participant’s home, or their local health clinic. Sessions were timed to coincide with a participant’s optimum performance during the drug cycle. If a speaker displayed signs of experiencing off periods the recording was interrupted and continued on a separate occasion. Data were collected on a Tascam DAT recorder (Tascam DA-P1), using a Beyerdynamic Microphone M58 placed around 30 cm from the participant’s mouth.

The current investigation focuses on a selection of tasks from the original study, namely one of the DDK tasks (repetition of “ba” for 5 s), and excerpts of the reading passage and conversational sample. The reading excerpt is represented in Table 2. The utterance structure represented here denotes the most common phrasing applied by the control speakers.

**Table 2:** Excerpt of the Reading Passage.

Utterance:	Number of syllables:
1. Yes thanks,	2
2. we went down town to see a film.	8
3. Do you have any plans for today yet?	10
4. (Yes)* I’ll be going to the exhibition in the Todd Centre.	15
5. Would you like to come as well?	7
6. Pam told my brother yesterday	8
7. that it is better than she expected.	10
8. Why don’t you meet me there at about two?	10
9. I would with pleasure	5
10. but I’m not sure whether I can join you.	10
11. I promised my nephew to take him to the zoo	12
12. to see the new camels and tigers that came in last week	14

* “Yes” in utterance 4 was excluded from analysis as speakers varied considerably in whether they placed a pause after this word and how long this was, which might have affected the rhythm measure.

This excerpt was chosen as it constituted a continuous sample of long and short utterances. It was taken from the middle of the reading passage to avoid the influence of initiation problems or unfamiliarity with reading aloud affecting the speaker’s performance. The sample added up to an average of 7.84 s articulation time (i.e. excluding pauses) for the control group, with a SD of 0.63 s.

In choosing appropriate sections of the conversational sample, it was important for the current investigation to arrive at a data set that was as comparable as possible to the read data. We therefore selected sections from the spontaneous speech task that resulted in a similar number of syllables, sample duration and variation in utterance length. Furthermore, it was essential that the conversational samples were comparable across speakers in order to be able to attribute any potential group differences to speaker performance rather than variations in speech material. For this purpose we performed a number of linguistic comparisons between the conversational data of the PwPD and control speakers. These were based on the results of a separate investigation into the language skills of these speakers (Ebert, 2015).

As part of this investigation, the recorded speech samples had been transcribed orthographically (this was possible with sufficient accuracy given the high level of intelligibility displayed by the selected PD speakers) and segmented into individual utterances. Utterances were defined as stretches of speech surrounded by pauses (defined acoustically, see below), irrespective of grammatical completeness. Unintelligible segments were included in the analysis as long as it was clear from the context which grammatical element they represented, e.g. “we went to XXX”, or “he left his XXX on the train”. If the unintelligible segment prevented us from reliably counting the number of words in the utterance or making grammatical judgements, the whole utterance was excluded from analysis. In line with the language acquisition literature, multiword nouns, most commonly place names such as Notre Dame, Whitley Bay, etc. were counted as single words, as they constituted single units of meaning. Phrases that did not contribute to the meaning of an utterance such as “you know, I mean” were classified as interjections and not considered in the analysis. Further exclusions came in the form of mazes and false starts (where participants corrected themselves within the same utterance), as well as disfluencies that resulted in whole or part-word repetitions. The remaining data were then analysed grammatically at phrase and clause level, investigating both length and complexity of output.

The statistical analysis (Mann-Whitney-U-Test) of linguistic features indicated no significant differences between the PwPD and control groups in the mean length of utterance (z = −0.787; p = .431; r = 0.13), or the percentage of grammatically complete utterances (z = −0.618; p = .536; r = 0.10), subordinate clauses (z = −0.169; p = .886; r = 0.03) and abandoned utterances (z = −0.309; p = .750; r = 0.05). The groups only differed in the percentage of mazes and disfluencies (z = −2.092; p = .036; r = 0.34), however, as these segments were excluded from the analysis, this fact was unlikely to influence the subsequent rhythm analysis. In addition to the linguistic analysis, we also compared the ratio of the number of vocalic to consonantal segments in the sample as this could impact on the rhythm analysis. This also showed no significant differences (z = −0.983; p = .326, r = 0.22) and the samples were thus deemed comparable.

### 2.3 Acoustic data analysis

For the purpose of the rhythm analysis, the selected reading and spontaneous speech extracts were analysed phonetically with the software Praat v 6.0.05 (Boersma & Weenink, 2016). False starts, mazes, disfluencies (i.e. words including phoneme repetitions and prolongations or repeated whole words), periods of speech freezing (i.e. abnormal prolongations of sounds within words) and filled pauses (i.e. vowel or nasal prolongations (“eh”, “em”) within or between utterances) were excluded from the sample. Unintelligible segments were also removed from the analysis. Vowel and consonantal intervals were then manually labelled on Praat tiers based on information in the oscillographic and spectrographic signals, following standard procedures described in the literature (see e.g. Grabe & Low, 2002; Liss et al., 2009; Low et al., 2000). Pauses were identified as periods of silence longer than 200 ms. Vocalic segments were defined as the interval between vowel onset and vowel offset, irrespective of the number of vowels included in the signal. They could thus include a single vowel, a diphthong, or two adjacent vowels from different words. The same applied to consonantal segments, i.e. they could include one or more consonants. As an example, the sequence “the importance” would thus have a CVCVCVC (“|th|e i|mp|o|rt|a|nce|”) structure following this labelling convention. Twenty percent of the data (one PwPD and one control speaker) were re-labelled by a separate examiner to ensure inter-rater reliability of the data. The results showed good agreement levels with a Crohnbach’s Alpha of 0.908 for the control participant and 0.960 for the PwPD.

Vocalic and consonant interval durations were subsequently extracted with a Praat script in order to calculate the rhythm metrics. Liss et al. (2009) investigated a large number of metrics which had previously been shown to capture rhythmic differences between languages (Low et al., 2000; Ramus et al., 1999; White & Mattys, 2007). Due the exploratory nature of this study, the same wide range of metrics was applied in the current analysis, namely the percentage of vowel versus consonant time in the signal (%V), the standard deviation of vowel and consonant duration (ΔV & ΔC), the pairwise variability index for vowels, consonants and vowel-consonant sequences (nPVI-V, rPVI-C, nPVI-VC & rPVI-VC) and the coefficient of variation for vowels, consonants and vowel-consonant sequences, (VarcoV, VarcoC & VarcoVC). Table 3 provides details on these measures based on Liss et al. (2009). In addition, a separate script extracted pause and speech durations as well as syllable numbers, in order to calculate articulation rate (i.e. the number of speech segments divided by articulation time excluding pauses), which was expressed in syllables per second.

**Table 3:** Description of rhythm metrics applied in this study (as detailed in Liss et al. (2009, p. 1339)).

Variable	Description
%V	Percent of utterance duration composed of vocalic intervals
∆V	Standard deviation of vocalic intervals.
∆C	Standard deviation of consonantal intervals.
VarcoV	Standard deviation of vocalic intervals divided by mean vocalic duration (×100).
VarcoC	Standard deviation of consonantal intervals divided by mean consonantal duration (×100).
VarcoVC	Standard deviation of vocalic + consonantal intervals divided by mean vocalic + consonantal duration (×100).
nPVI-V	Normalized pairwise variability index for vocalic intervals. Mean of the differences between successive vocalic intervals divided by their sum (×100).
rPVI-C	Pairwise variability index for consonantal intervals. Mean of the differences between successive consonantal intervals.
nPVI-VC	Normalized pairwise variability index for vocalic + consonantal intervals. Mean of the differences between successive vocalic + consonantal intervals divided by their sum (×100).
rPVI-VC	Pairwise variability index for vocalic and consonantal intervals. Mean of the differences between successive vocalic and consonantal intervals.

For the DDK analysis, syllable durations were defined as the interval from the release of one stop closure to the release of the next as the associated bursts constituted the most reliable measurement point in the data. The mean syllable duration (in ms), as well as its standard deviation (SD) and coefficient of variation (CoV) were subsequently extracted for each speaker. These measures represent standard measures applied to DDK tasks in the literature, and reflect the perceptual concepts of speed and regularity of repetition. Whilst it would have been possible to submit the DDK data to the same rhythm metrics as the connect speech material in order to detect any potential relationships, the reason for including these tasks was to investigate to what degree a simple perceptual clinical evaluation of DDK performance could inform on the level of rhythmic impairment in an individual. The analysis therefore focused only on rate and regularity of production rather than the more complex rhythm measures.

### 2.4 Statistical analysis

In order to address the three research questions, a number of comparisons were performed. Questions 1 and 2 were answered by investigating potential differences between groups. This would highlight whether any performance change could be picked up by the measures in the mild speaker group, and, in addition, whether these were evident across all tasks. We also conducted a comparison of task differences between reading aloud and spontaneous speech for each of the speaker groups. This was based on previous reports of speakers with dysarthria not being able to effect the same changes between these tasks as healthy controls. This comparison was therefore used to provide further information on whether spontaneous speech would be a suitable medium to assess rhythmic performance. The final question was addressed by correlating the rhythm results from the reading and monologue task with the speakers’ DDK performance variables.

The statistical analysis was conducted with IBM SPSS version 22. Due to the high number of rhythm variables investigated for the group comparison, it was not feasible to compare individual parameters without introducing a Type I error. A Bonferroni correction would have resulted in extremely low alpha levels which would have made it difficult to detect any differences with the small number of speakers included in this study. For this reason it is not advised for exploratory studies such as the current. An ANOVA was not appropriate either as the data were not normally distributed, and in any case this would have resulted in a high number of post-hoc tests being performed. Two analyses that lend themselves to differentiating groups on the basis of multiple parameters are Principal Component Analysis (PCA) and Discriminant Analysis. PCA results in a number of components which are linear combinations of the original independent variables, however, it does not highlight which parameters were involved in the differentiation.

As we aimed to also identify to what degree the two speech tasks differed in relation to which rhythm metric could distinguish the groups, we opted for a discriminant analysis instead, in line with Liss et al.’s (2009) methodology. A univariate discriminant analysis was conducted first at the 5% significance level to reduce the number of variables tested in the multi-variate discriminant analysis. Only those variables deemed to be significant (p < .05) in the univariate analysis were taken forward to the multivariate analysis where all independents were entered together. Separate analyses were run for the reading aloud and spontaneous speech tasks for this purpose. The DDK task only included three variables, and a discriminant analysis was therefore not appropriate. In this case, a Mann-Whitney-U-Test was performed, with an alpha level of p < .05.

To investigate the task differences, only those variables that were able to differentiate the groups from each other in the reading aloud task were used. This reduced the number of variables from eleven to five. Keeping in mind that the data were not normally distributed, the non-parametric Wilcoxon Signed Rank Test was used to determine differences between reading aloud and spontaneous speech within each of the speaker groups, again applying an alpha level of p < .05. Bonferroni corrections were not applied for the reasons given above, however, this fact is considered in the discussion of the results.

In keeping with the non-parametric choice of tests, Spearman’s rho was applied to investigate relationships between reading aloud, spontaneous speech and DDK performance.

## 3 Results

**Fig. 1:**
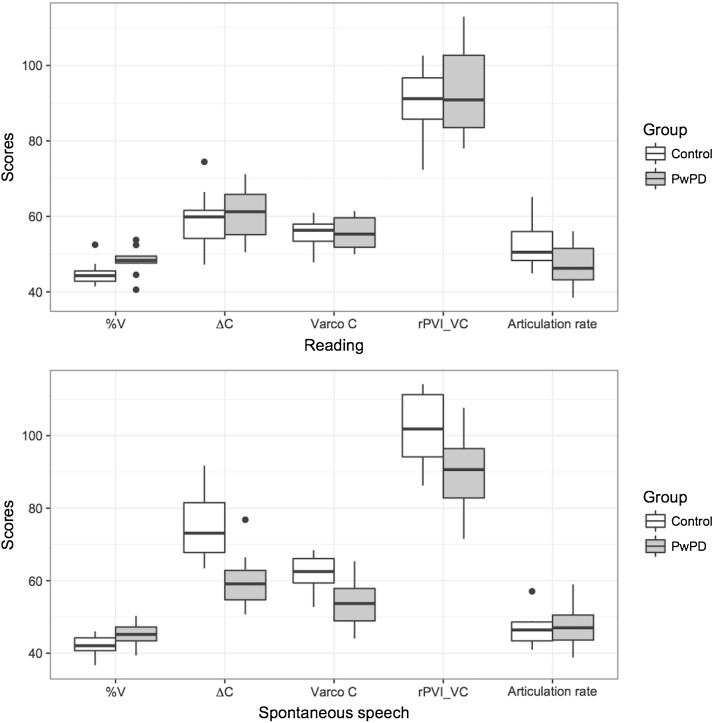
Performance of PwPD and Control Speakers for the parameters included in the discriminant analysis for reading aloud (a) and spontaneous speech (b). Please note that values for articulation rate have been upscaled in order to fit the figure. Values should be divided by 10 in order to arrive at the proper value, i.e. a rate indicated as 50 on the figure constitutes an actual rate of 5 syll/s. The data presents the median, first and third quartiles, as well as outliers (defined as points whose value lies outside 1.5 times the interquartile range).

As the current participant group is different to the original study the data were collected for, all results reported here are unique to this paper. Table 4 presents the descriptive results for the three tasks under investigation and Fig. 1 furthermore highlights the group differences across reading aloud and spontaneous speech for the parameters that were included in the discriminant analysis. Table 5 summarises the statistical findings.

**Table 4:** Data Descriptives (group means and SD) for PwPD and Control Speakers for Reading Aloud and Spontaneous Speech (significant parameters only) and DDK Tasks.

Data Descriptives								
	PwPD				CON			
*Variable*:	*Reading Aloud*		*Spont. Speech*		*Reading Aloud*		*Spont. Speech*	
	*mean*	*SD*	*mean*	*SD*	*mean*	*SD*	*mean*	*SD*
**Rhythm:**								
%V	48	3.7	45	3.2	45	3.2	42	2.9
ΔC	60	6.8	60	7.6	59	7.7	75	9.6
VarcoC	56	4.4	54	6.3	55	4.3	62	5.4
rPVI-VC	93	11.7	89	10.8	90	8.8	102	10.2
Rate	4.7	0.6	4.8	0.6	5.3	0.7	4.7	0.5

DDK:	PwPD		CON	
	*mean*	*SD*	*mean*	*SD*
Mean syll dur in ms	185	32	146	13
SD of syll dur in ms	20	8	11	3
CoV of syll dur in ms	107	42	73	17

**Table 5:** Statistical Analysis Results for the Significant Parameters of the Discriminant Analysis, as well as Subsequent Analysis Parameters for Task Comparison (Reading Aloud versus Spontaneous Speech in PwPD and control speakers (CON)) and DDK.

Independent variable	Standardised coefficients	Discriminant loading (rank)	F	p
**Reading Aloud**				
%V	0.425	0.856 (2)	4.659	0.045
Rate	0.673	0.945 (1)	5.677	0.028
Group centroid (PwPD)	0.564			
Group centroid (CON)	−0.564			
Wilkes lambda	0.739			0.045
(Canonical correlation)^2	0.261			

**Spontaneous Speech**				
%V	−0.746	−0.407 (5)	5.473	0.031
ΔC	0.867	0.693 (1)	15.900	0.001
rPVI-C	−0.473	0.513 (3)	8.677	0.009
Varco C	0.720	0.533 (2)	9.419	0.007
rPVI-VC	−0.101	0.458 (4)	6.936	0.017
Group centroid (PwPD)	−1.290			
Group centroid (CON)	1.286			
Wilkes lambda	0.352			0.006
(Canonical correlation)^2	0.648			

Task Comparison: Reading Aloud vs. Spontaneous Speech						
	PwPD			CON		
Variable	z	r	p	z	r	p
%V	−2.090	0.467	.037	−2.293	0.513	.022
ΔC	−0.255	0.057	.799	−2.497	0.558	.013
VarcoC	−0.833	0.186	.386	−2.293	0.513	.022
rPVI_VC	−1.682	0.376	.093	−2.191	0.490	.028
Rate	−0.833	0.186	.386	−2.090	0.467	.037

Group Comparison DDK Task: PwPD vs. CON			
Variable	z	r	p
Mean	−2.721	0.608	.007
SD	−2.950	0.660	.003
CoV	−1.965	0.439	.049

### 3.1 Group differences

The univariate analysis identified two variables that appeared to be significant predictors of group for the reading task, %V (p = .045) and rate (p = .028). For spontaneous speech, the univariate analysis identified five variables that appeared to be significant predictors of group: ΔC (p = .001), %V (p = .031), rPVI-C (p = .009), Varco-C (p = .007), and rPVI-VC (p = .017). On this basis, the multivariate discriminant analysis focused on six of the original eleven variables, i.e.%V, ΔC, Varco-C, RPVI-C and rPVI-VC. The results confirmed those for the univariate analysis (see Table 5 for details). For reading aloud, %V and rate were significant predictors of group. Together these variables correctly classified 70% of cross validated cases (applying the leave one out method). In spontaneous speech, %V, ΔC, rPVI-C, VarcoC and rPVI-VC were all significant predictors of group and together they were able to correctly classify 70% of cross validated cases. rPVI-C was highly correlated with ΔC and rPVI-VC (r = 0.909 and 0.896, respectively). When this parameter was excluded from the analysis, the model’s ability to correctly classify individuals improved to 80%. In all cases, the control speakers were situated more towards the stress-timed end of the continuum, e.g. they had higher PVI or lower%V values. In relation to rate, the PwPD tended to speak at a slower rate than the healthy controls.

The DDK task analysis indicated statistically significant differences with large effect sizes (Cohen, 1988) across all three measures. The PD group showed longer mean syllable durations, i.e. a slower repetition rate (p = .007). Durational variability was also significantly higher in the PD group. This was particularly prominent for the SD of syllable duration (p = .003), whereas the CoV analysis showed lower levels of significance (p = .049), suggesting that the increases in variability were partly caused by the slower repetition rate.

### 3.2 Differences between reading aloud and spontaneous speech

Based on the results of the discriminant analysis, only five variables were pursued for further analysis in the task comparison, namely%V, ΔC, VarcoC, rPVI-VC and rate. Statistical results highlighted differences between reading aloud and spontaneous speech in both the PD and the control group. However, the PwPD only showed these changes in one measure (%V, see Table 4), whereas the control data resulted in significant differences across all five rhythm metrics (i.e.%V, ΔC, VarcoC, rPVI-VC as well as rate). Results for the rhythm metrics suggested that the spontaneous speech task was produced with a more stress-timed rhythm across both groups. In relation to rate, the control participants spoke significantly slower in this task. All significant results were associated with large effect sizes (Table 5).

### 3.3 Relationship between tasks

Table 6 summarises the statistical results obtained from the correlation analysis between the four rhythm variables determined by the discriminant analysis, articulation rate and the three DDK measures. For both groups there were some significant relationships between the rhythm metrics, although there was no consistency as to which variables were related to each other. There was also some relationship between articulation rate and some of the rhythm metrics in reading aloud and spontaneous speech across the two groups, mostly with ΔC and rPVI-VC. In each case this correlation was negative, indicating that a slower rate resulted in higher variability levels. The PwPD were the only group to show correlations between their DDK and speech performance. Specifically, the standard deviation of syllable durations in the DDK task correlated with ΔC (in spontaneous speech), as well as with articulation rate in both tasks. The coefficient of variation also correlated with rate in spontaneous speech. The mean syllable repetition rate, on the other hand, was not predictive of any of the variables taken from the speech tasks.

**Table 6:** Results for Correlations between Variables for the PwPD and the Control Speakers.

## 4 Discussion

This study investigated whether acoustic rhythm metrics were sensitive to subtle performance changes as shown by speakers with mild hypokinetic dysarthria, whether such changes could be demonstrated in reading aloud as well as a more naturalistic spontaneous speech task, and whether a simple DDK analysis could reflect similar motor deficits as the more complex rhythm metrics applied to the speech tasks.

To answer the first question, this study was able to highlight differences between the PwPD and the control group on the basis of a number of rhythm metrics. The%V measure consistently differentiated the groups across both tasks. Furthermore, there was an indication that some of the consonant based metrics (ΔC and VarcoC) captured further differences in speech performance in spontaneous speech, in addition to the syllable based metric rPVI-VC. Although at least some of these results need to be interpreted with caution due to the relatively high p-values associated with them, they are to some degree comparable with those of Liss et al. (2009) who also identified VarcoC, and%V amongst the most discriminatory metrics between their dysarthric and control groups. Liss et al. (2009) further noted that the%V measure had also been judged as highly discriminatory and robust against articulation rate changes in cross-linguistic research in healthy speakers (White & Mattys, 2007). Based on the evidence available from this and previous studies, one could thus conclude that the%V measure is a consistent and sensitive metric to differentiate impaired and healthy speaker populations across a number of speech tasks and severity levels.

The question that follows on from this observation is why%V is particularly sensitive to changes in speech timing observed in dysarthria. In the current study, the PD group had a higher percentage of vowel time compared to the healthy controls across both speech tasks. This could be indicative of a problem in reducing normally unstressed vowels. This feature was first described by Leuschel and Docherty (1996) who, in the absence of the rhythm metrics that are available now, expressed this problem in terms of a lower percentage of (perceptually identified) reduced vowels in their speakers’ sample. As indicated above, this was subsequently confirmed by Liss et al. (2009) through their acoustic rhythm metrics. However, if vowel reduction were a problem, one might also have expected the other vowel metrics (ΔV or VarcoV) to highlight group differences, as had been the case in Liss et al.’s (2009) study. That is, if unstressed vowels are not reduced to the same to degree as in healthy speakers, one would expect a smaller amount of variability in vowel duration. This was not the case here.

Another explanation could be that the relationship between vowels and consonants was altered whilst the vowel to vowel timing was preserved, thus resulting in the altered consonant and syllable based metrics. This could be due to changes in rate, which affects vowel duration to a greater degree than consonants (Duez, 1999; Gay, 1981; Tjaden & Turner, 2000). However, the statistical data suggest that there were no significant group differences in articulation rate in the spontaneous speech task, despite the observed difference in consonant metrics. A further possibility might thus be that consonants in themselves were affected, at least in the spontaneous speech sample. It is not possible to confirm the underlying reason for this result without a detailed phonetic analysis of the participants’ speech performance, but it would be interesting to examine whether subtle signs of articulatory undershoot or a slowness in articulatory movement such as previously reported by Ackermann, Gröne, Hoch, & Schönle (1993), Ackermann and Ziegler (1991), Forrest, Weismer, & Turner (1989) or Skodda, Grönheit et al. (2010) possibly led to inappropriate shortening or lengthening of consonantal segments, consonant elision in clusters, or other articulatory changes leading to alterations in the timing of consonantal segments. The kinematic literature also contains some interesting indications that could be followed up in future research with regard to their impact on speech rhythm, such as Forrest et al.’s (1989) findings that vowel durations were reduced whilst VOT was increased in their speakers with PD, thus altering the normal relationship between vowels and consonants. Ackermann & Ziegler’s (1991) observation that the degree of undershoot was determined by the stress status of a syllable might also be a potential feature contributing to the current findings.

To answer our second question, whether spontaneous speech samples lend themselves to capture disturbances of speech timing, it was interesting to note that more group differences could be detected in the spontaneous sample than in reading aloud. This suggest that spontaneous speech was able to differentiate between speaker groups as well as reading aloud, if not better. Whilst one has to be careful again in the interpretation of this result due to the lack of the Bonferroni correction, the large effect sizes associated with the significant results provide some support that these differences are real. In addition, the relatively consistent performance within each group, showing changes across all variables for the control speakers and no changes except for one variable for the PwPD provide a further level of credibility to the difference in patterns observed across the two groups.

Aside from the statistical issues, one could argue that the finding that spontaneous speech was more suited to detect group differences than reading aloud was primarily due to articulation rate characteristics rather than rhythmic behaviour in itself. In other words, the presence or absence of rhythmic changes across reading aloud and spontaneous speech might have coincided with the rate characteristics of each task which showed differences for the control group but not the PwPD. However, as discussed above, %V is seen as being robust to rate, yet still differed both across tasks and between groups. In addition, no consistent correlation between rate and the remaining rhythm metrics could be observed in support of this point with the exception of rPVI-VC which correlated significantly with rate in both tasks for both groups.

Finally, one might argue that the results were simply due to differences in the nature of the speech material rather than reflecting speaker behaviour. However, several aspects speak against this point. First of all, the linguistic analysis suggested no significant differences in output structure between the groups, and whilst no detailed analysis was performed of the exact phonotactic structure, there is no specific reason why all control participants or all PwPD should have produced monologues similar to their group members but different to the other group. In addition, the speakers differed in the%V measure across both the spontaneous and the identical reading aloud samples, which is one of the metrics suggested to pick up on phonotactic differences (Ramus et al., 1999). The current results can therefore be argued to reflect a genuine difference in speech performance instead of variations in the phonetic make-up of the material.

One area that does require further investigation though relates to the earlier observation that the rhythm metrics investigated in this paper focus exclusively on speech timing and ignore other influencing variables such as pitch and loudness performance. As such, it needs to be established to what degree the differences observed in this study are inherent to a problem with rhythm that can be attributed to a disturbance of the basal ganglia, as proposed by e.g. Skodda, Flasskamp et al. (2010), Skodda, Grönheit et al. (2010), or are simply a reflection of impaired articulation patterns. Lowit’s (2014) discussion of the potential dissociation between metric results and speech production patterns of individuals with dysarthria supports the need for further investigations of speaker groups with distinct neurological aetiologies on the basis of detailed segmental analyses in order to shed more light on this question.

There are no previous reports on the rhythmic performance of speakers with speech impairments in spontaneous speech, and the current results can therefore not be compared to the existing literature. However, there are previous studies on differences between reading aloud and spontaneous speech that can put the current results into context. The results suggest that the PD group showed fewer differences between reading aloud and spontaneous speech than the control speakers across the various rhythm metrics, i.e. their speech production was more rigid than that of their healthy counterparts and they did not change their speech performance between the tasks. This has already been reported by Leuschel and Docherty (1996) in a comparison of rate, intensity and F0 performance in reading aloud and spontaneous speech, as well as Lowit et al. (2006) in relation to the ability to change articulation rate in a sentence reading task. It also sits well with Dromey et al.’s work on dual tasking which clearly shows that speech performance deteriorates when cognitive load increases (Dromey & Bates, 2005; Dromey & Benson, 2003; Dromey & Shim, 2008), as well as Huber & Darling’s (2011) findings of greater difficulties in coordinating language planning and respiratory support in monologues than in reading in their PwPD. The fact that more group differences were detected in the spontaneous speech task points to the fact that the task demands might have been higher in that task and speakers were no longer able to replicate the performance of the healthy controls. Research by van Lancker et al. (2012) reports similar task effects on the speech of an individual with PD in relation to speech fluency.

The final question related to whether DDK performance would reflect similar deficits of speech timing, thus avoiding the need to conduct a time consuming rhythm analysis in clinical contexts. Although there were some significant correlations between speech and DDK tasks identified, overall, the current study suggests that the answer is negative. There were no correlations between any of the DDK measures and speech performance in the control group. In the PD group, only ΔC in spontaneous speech correlated with the SD and CoV in the DDK tasks, and the remaining correlations occurred between SD in DDK and rate rather than further rhythm measures. In addition, the relationship between ΔC and SD DDK cannot be interpreted easily, as it was of a positive nature, i.e. higher variability levels of syllable repetitions correlated with higher ΔC values in spontaneous speech. This is problematic as an increase in variability in DDK syllable repetitions is indicative of a reduction in movement control and thus a disorder, whereas higher ΔC values in speech tasks indicate a more stress-timed rhythm in English language contexts and are thus a sign of a more normal performance. The results for the two tasks in the current group thus contradict each other.

Overall, our findings leave us with the conclusion that DDK tasks appear to be useful in highlighting underlying problems with speech motor control, based on the strong group statistics yielded by both mean repetition rate and its standard deviation in the group comparison. However, there was little evidence that the DDK performance was predictive of rhythmic behaviour in either group, i.e. irregularities in syllable repetition during DDK tasks did not necessarily indicate a disturbance of rhythm during speech. This is in line with the most recent findings by Staiger, Schölderle, Brendel, Bötzel et al. (2017), Staiger, Schölderle, Brendel, & Ziegler (2017) that DDK performance showed little relationship to a comprehensive speech assessment of patients with dysarthria, thus demonstrating yet again that clinicians need to be careful on how they interpret the results of these tasks.

As already alluded to above, one has to apply a degree of caution to the interpretation of the above results as the investigation only included 10 speakers in each group, which lessens the predictive power to some degree. In particular, one could assume that more correlations might become significant, or more rhythm measures show group or task differences as participant numbers increase. On the other hand, our statistical results on the whole were clear cut, i.e. they showed low p-values and high effect sizes for the significant results, and high p-values and low effect sizes for the non-significant results. There were thus no borderline cases where one could have hypothesized that results were likely to change with the inclusion of a few more speakers, although there is of course always potential for this to happen. We also found clear distinctions in relation to the number of observed differences between groups or tasks. And whilst far from ideal, our group size compares favourably to the key publications in the existing literature in rhythm research (e.g. four participants in Ramus et al. (1999), six in White and Mattys (2007), and eight to twelve per group in Liss et al. (2009)), suggesting that it is possible to answer questions reliably with smaller speaker groups in this field. One should also not lose sight of the fact that the current PD group only included speakers with mild levels of dysarthria who could be expected to perform within or close to the normal range for at least some measures. Finding significant differences in this context is thus additionally suggestive that our variables highlighted areas of difficulty for these speakers. Nevertheless, it is clear that further research with larger numbers of participants needs to be performed in order to validate the claims made in this study.

## 5 Conclusion

This paper makes several important contributions to rhythm research in dysarthria. It is the first to highlight the fact that it is possible to use conversational data to detect rhythmic differences in clinical speaker groups, and more importantly that this speech task is potentially more sensitive to speech problems in speakers with mild hypokinetic dysarthria than reading data. The fact that speech differences could be picked from data samples that did not match exactly in content is an important finding as it allows future pooling of data from various sources to allow a more large scale assessment of rhythm as long as similarity checks are performed. This might help to address the paucity of studies into speech rhythm in dysarthria to at least some degree.

Secondly, this paper was able to demonstrate the highly sensitive nature of rhythm metrics to the underlying neurological disorder and the ensuing importance of these tools for research purposes, both as diagnostic and outcome measures. As a next step, secondary data analyses as postulated above could help to confirm this fact, widen the analysis to other types and severities of dysarthria and allow researchers to establish the metrics’ power to monitor performance change, thus paving the way for them to become a more sensitive outcome measure than some of those currently available. Such research would serve to confirm which of the many metrics available at the moment are most consistent in highlighting timing deficits common to dysarthria. In light of the fact that DDK tasks were not able to clearly reflect the speech timing deficits identified by the rhythm metrics, the use of the latter as diagnostic or outcome measures is currently limited to the research field, given the necessary speech analysis skills and time consuming nature of the procedure. However, research is currently underway to establish more reliable automatic recognition and parsing systems that might in future simplify speech analysis sufficiently to allow clinical application of these metrics. The fact that the rPVI-VC was one of the measures that could distinguish the groups from each other is particularly promising in this regard, as it will be easier for automated systems to parse signals for syllables than for individual vowels and consonants. However, further research into the suitability of this parameter is required due to its observed relationship with articulation rate.

In conclusion, this paper adds to the emerging literature on rhythm in disordered populations by providing data on a novel speaker group, i.e. those with very mild impairment, and by demonstrating that rhythm analysis can be applied to less structured data than has previously been thought. This opens up a wide range of opportunities for the use of rhythm metrics in future investigations of speakers with dysarthria.

## Footnotes

1 Irrespective of the above discussion about whether the measures under investigation here do in fact reflect the rhythm or simply the timing of speech, we will retain the term “rhythm metric” in alignment with the current literature.
